# Assessment of a Community-Based Exercise Program for Older Adults in a Mixed Rural/Urban Catchment Area: Silver Sneakers in Central Pennsylvania

**DOI:** 10.5888/pcd19.210283

**Published:** 2022-01-27

**Authors:** Eileen Flores, Sage Nakagawa, Robinn Moyer, Shirley M. Bluethmann

**Affiliations:** 1 Pennsylvania State University College of Medicine, Department of Public Health Sciences, Hershey, Pennsylvania; 2 Queen’s University, Kingston, Ontario, Canada; 3 Penn State Cancer Institute, Hershey, Pennsylvania

## Abstract

The purpose of our study was to understand the capacity of Silver Sneakers, a federally funded and community-based exercise program, to serve older adults (aged ≥65 years) in our mixed rural/urban catchment area of central Pennsylvania. We identified 139 registered Silver Sneakers program locations; of these, 18 were closed because of the COVID-19 pandemic. We used questionnaires to interview Silver Sneakers program staff by telephone (n = 80 of 121, response rate of 66%). Most programs were offered by private gyms (52%). Fewer programs were in rural counties than in urban counties. Most facilities reported that membership was equally mixed by gender, and member retention strategies included program perks and promotion of Silver Sneakers as a Medicare benefit. Most (89%) programs were able to continue classes during the pandemic, in part by adapting to video platforms. Overall, Silver Sneakers programs offer a sustainable option to facilitate access to exercise programs and reduce barriers to physical activity among older adults in our catchment area.

**Summary**

**What is already known on this topic?**

Silver Sneakers is an exercise program widely available in the US and free to many with Medicare benefits. Little data exist about availability of programs in rural or urban communities or specific data about which classes are available, especially in mixed rural/urban regions.

**What is added by this report?**

Our study provides a comprehensive assessment of Silver Sneakers programs accessible to older adults (aged ≥65 years) in the mixed rural/urban catchment area of the Penn State Cancer Institute.

**What are the implications for public health practice?**

The results of this study will provide contextual data for similar mixed urban and rural regions looking to leverage this health care resource, especially for areas serving traditionally underserved regions with high numbers of older adults.

## Objective

The purpose of our study was to understand the capacity of Silver Sneakers, a community-based and free-to-most exercise program, to serve older adults (aged ≥65 years) in our mixed rural/urban catchment area of the Penn State Cancer Institute in central Pennsylvania. Although regular exercise reduces disease risk and promotes healthy aging (1–3), many older adults do not achieve an adequate amount of exercise in their daily lives because of barriers such as cost, fear of injury or pain, lack of motivation, and lack of transportation (4–9). Silver Sneakers programs are available to most Medicare beneficiaries and address these barriers, but little data are available about the programs in mixed rural/urban regions.

## Methods

We performed an observational study, with data collection and analysis occurring between August 2019 and January 2021. First, we identified all registered Silver Sneakers program locations in our 28-county catchment area. Second, we conducted a one-time telephone questionnaire with Silver Sneakers program staff.

To identify all registered Silver Sneakers program locations in our catchment area, we manually matched geographic information system data (based on zip codes) with locations on the Silver Sneakers website in the 28-county catchment area by using their web-based locator tool (10). By using information provided on the website, we characterized program details by location and facility type and later verified these details as part of our questionnaire. Additionally, we compiled regional descriptors to provide context to the catchment area environment, including population demographics and rurality designation by county.

Next, a study team member (R.M.) called each Silver Sneakers facility and invited a program staff member to participate in a 20-minute telephone questionnaire. If they agreed to participate, an interview consisting of 21 multiple-choice questions was then administered to assess member and program characteristics, training of staff, and program marketing. For open-ended responses (eg, “other”), the interviewer requested the participant to elaborate.

Quantitative and qualitative responses to the telephone questionnaire were manually captured by the interviewer and recorded in a secured program database. Quantitative data were cleaned and summarized using descriptive statistics. Responses to each question were reported as the number and percentage per total available responses for each question, and characterized by geographic area type (ie, urban or rural). Qualitative data (ie, open-ended responses) were used to complement or clarify responses on the questionnaire regarding program characteristics and other details.

The Silver Sneakers study protocol was reviewed by the Institutional Review Board at the Pennsylvania State University College of Medicine and deemed exempt from full board review.

## Results

In 27 of 28 counties, 139 programs were identified. The mean number of Silver Sneakers locations was 4.96 per county (range: 0–18). Berks, Lancaster, Dauphin, and York counties reported the highest program densities, between 14 and 18 locations per county. These urban counties encompass or border the larger metropolitan areas of Harrisburg and Philadelphia, Pennsylvania, and Baltimore, Maryland (Figure).

**Figure:**
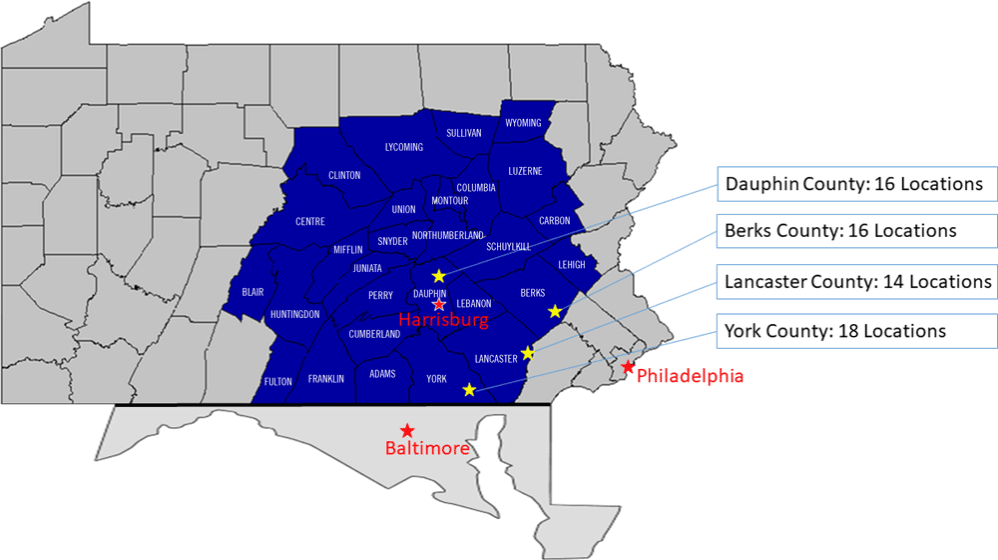
Central Pennsylvania counties with high numbers of Silver Sneakers locations, 2019–2021.

Most programs were offered by private gyms (72 [52%]) (eg, Gold’s Gym). Others were offered by YMCA (41), community centers (18), residential care (4), and senior centers (4). Fewer Silver Sneakers programs were found in counties designated as rural (11) when compared with urban areas with higher population density (Table). Of all Silver Sneakers programs, 35% were in 20 rural counties and 65% were in 8 urban counties. The proportion of older adults living in each county was less than 20% in urban areas and generally 20% or greater in rural areas. Sullivan County, a rural county of 6,071 residents, was the only county without a Silver Sneakers program in the catchment area, yet it also had the highest proportion of seniors (28%) compared to other counties (Table).

**Table:** Silver Sneakers Locations and County Population Descriptors, Pennsylvania, 2019–2021

Central Pennsylvania counties	No. of Silver Sneaker locations	County population^a^	Percentage of population aged ≥65 years	Proportion of Silver Sneakers programs by population aged ≥65 years (per 10,000)	Rural or urban
**Adams**	2	102,811	20.4	1	Rural
**Berks**	16	420,152	17.2	2	Urban
**Blair**	8	122,492	20.8	3	Rural
**Carbon**	1	64,227	21.2	1	Rural
**Centre**	3	162,805	14.2	1	Rural
**Clinton**	1	38,684	18.7	1	Rural
**Columbia**	4	65,456	19.5	3	Rural
**Cumberland**	6	251,423	18.5	1	Urban
**Dauphin**	16	277,097	17.0	3	Urban
**Franklin**	5	154,835	19.6	2	Rural
**Fulton**	1	14,523	21.6	3	Rural
**Huntingdon**	1	45,168	20.7	1	Rural
**Juniata**	1	24,704	20.1	2	Rural
**Lancaster**	14	543,557	17.9	1	Urban
**Lebanon**	2	141,314	19.4	1	Urban
**Lehigh**	9	368,100	16.7	1	Urban
**Luzerne**	9	317,646	19.9	1	Urban
**Lycoming**	4	113,664	19.3	2	Rural
**Mifflin**	1	46,222	21.7	1	Rural
**Montour**	1	18,240	21.0	3	Rural
**Northumberland**	3	91,083	21.4	2	Rural
**Perry**	1	46,139	18.5	1	Rural
**Schuylkill**	5	142,067	20.4	2	Rural
**Snyder**	2	40,540	19.0	3	Rural
**Sullivan**	0	6,071	28.3	0	Rural
**Union**	3	44,785	18.1	4	Rural
**Wyoming**	2	27,046	21.2	3	Rural
**York**	18	448,273	17.4	2	Urban

a Source: US Census Bureau (12).

Eighteen gyms closed during the COVID-19 pandemic, leaving a sample of 121 participating facilities. Of the 121 facilities, 80 agreed to participate in the questionnaire. Among the response categories provided, most facilities (69%) reported membership was equally mixed by gender, and 94% reported that their members ranged from 65 to 80 years old. Program staff said that many members exercised several times per week with friends or family. Additionally, 35% reported that social opportunities were a primary reason that participants remained active in Silver Sneakers. Membership retention strategies included program perks (64%) and promotion of Silver Sneakers as a Medicare benefit (11.3%). Classic class (ie, low-impact aerobics) was the most popular class (83.8%). Most (89%) of the facilities reported that they were still able to offer Silver Sneakers during the pandemic, with 60% reporting that they were able to adapt the program format to video platforms to conduct classes.

## Discussion

Silver Sneakers programs provide a sustainable option to facilitate access to exercise programs and increase physical activity among older adults nationwide. To the best of our knowledge, this is the first evaluation study to provide a comprehensive assessment of Silver Sneakers programs accessible to older adults in the Penn State Cancer Institute catchment area, or others in the central Pennsylvania region.

Our assessment highlighted an opportunity to maximize the use of memberships by expanding programs within rural communities in our area. Fewer programs and a higher proportion of older adults were present in rural compared with urban areas, which generally have more economic output. Consideration of programs and infrastructures in areas where older adults reside may be beneficial, particularly in areas that have high populations of older adults but few or no programs available.

Our results also suggested that offering program perks that promoted social gatherings seemed more relevant to older adults compared with other strategies. Older adults were mostly characterized as sociable, indicating that the environment fostered friendships and maintained their interest.

The main limitations of our study were its cross-sectional nature and the timing of the COVID-19 pandemic during our data collection period. Despite these limitations, our questionnaire had a high response rate (66%). Silver Sneakers continued in 89% of the facilities, which speaks to its high adaptability to change. Common barriers related to access, cost, technology, and transportation were exacerbated for older adults during the pandemic but highlighted opportunities to improve with online technology, which suggests that it may play an integral role in future exercise instruction (13,14).

Providing access to programs such as Silver Sneakers is a step toward helping older adults meet recommended exercise guidelines. Our study provides contextual and relevant data for mixed rural/urban regions looking to leverage this health care resource, especially for areas serving traditionally underserved regions with high numbers of older adults.
